# Supplementary material for: Identification of photoperiod-induced specific miRNAs in the adrenal glands of Sunite sheep (Ovis aries)
Source: Front Vet Sci. 2022 Jul 22;9:888207. doi: 10.3389/fvets.2022.888207 (PMC9354845; doi:10.3389/fvets.2022.888207)
Supplement: Supplementary file 1 [file Data_Sheet_1.ZIP › Supplementary file/miRDeep2 file.zip!/miRDeep2 file/Know_Structure/SPLP42A1/oar-let-7c.pdf]

miRBase precursor : oar-let-7c
Total read count : 109810
oar-let-7c read count : 109746
remaining reads : 64

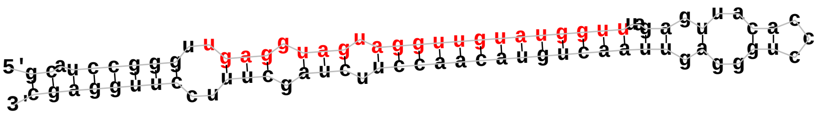

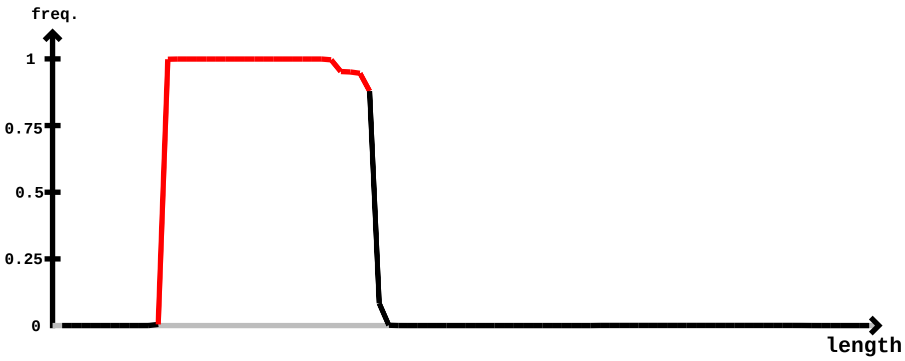


```
           oar-let-7c
5'- gcauccgggu ugagguaguagguuguaugguu uagaguuacacccugggaguuaacuguacaaccuucuagcuuuccuuggagc   -3'    exp
    ((.((((((..(((.(((.(((((((((((((..((.(..((...))..).))))))))))))))).))).)))..))))))))   reads      mm       sample
    ........guugagguaguagguuguauggu.....................................................   5          0        seq
    ........guugagguaguagguuguaugguu....................................................   4          0        seq
    ........guugagguaguagguuguauAguu....................................................   1          1        seq
    .........uugagguaguagguuguaug.......................................................   2          0        seq
    .........uugagguaguagguuguaugg......................................................   12         0        seq
    .........uugagguaguagguuguauAg......................................................   1          1        seq
    .........uugagguaguagguuguauAgu.....................................................   4          1        seq
    .........uugagguaguagguuguauggu.....................................................   131        0        seq
    .........uugagguaguagguuguauAguu....................................................   4          1        seq
    .........uugagguaguagguuguaugguu....................................................   294        0        seq
    .........uugagguaguagguuguaugguuu...................................................   21         0        seq
    .........uugagguaguagguuguauAguuu...................................................   1          1        seq
    ..........ugagguaguagguuUua.........................................................   1          1        seq
    ..........ugagguaguagguugua.........................................................   281        0        seq
    ..........ugagguaguaAguugua.........................................................   10         1        seq
    ..........ugagguagGagguugua.........................................................   37         1        seq
    ..........uUagguaguagguugua.........................................................   1          1        seq
    ..........ugaAguaguagguugua.........................................................   2          1        seq
    ..........ugagguaguagguuguau........................................................   4342       0        seq
    ..........ugagguaguagguuUuau........................................................   13         1        seq
    ..........ugaAguaguagguuguau........................................................   35         1        seq
    ..........ugagguaguaAguuguau........................................................   18         1        seq
    ..........ugagguagGagguuguau........................................................   402        1        seq
    ..........ugagguUguagguuguau........................................................   2          1        seq
    ..........ugagguaguagguuUuaug.......................................................   1          1        seq
    ..........ugagguaguagguuguaug.......................................................   178        0        seq
    ..........ugagguaguagguuguaugg......................................................   522        0        seq
    ..........ugagguaguagguuguauggu.....................................................   7191       0        seq
    ..........ugagguaguagguuguaugguu....................................................   86998      0        seq
    ..........ugagguaguagguuguaugguuu...................................................   9021       0        seq
    ..........ugagguaguagguuguaugguuua..................................................   38         0        seq
    ..........ugagguaguagguuguauAguuua..................................................   81         1        seq
    ..........ugagguaguagguuguaugguuuag.................................................   3          0        seq
    ...........gagguaguagguuguau........................................................   1          0        seq
    ...........gagguaguagguuguauggu.....................................................   4          0        seq
```

oar-let-7c

```
gcauccggguugagguaguagguuguaugguuuagaguuacacccugggaguuaacuguacaaccuucuagcuuuccuuggagc
...........gagguaguagguuguaugguu....................................................   71   0   seq
...........gagguaguagguuguaugguuu...................................................   9    0   seq
............agguaguagguuguaugguu....................................................   4    0   seq
................................uagaguuacacccugggagu................................   2    0   seq
................................uagaguuacacccugggaguu...............................   3    0   seq
....................................................uaacuguacaaccuucuagcuuuc........   1    0   seq
.....................................................aacuguacaaccuucuagcuuu.........   2    0   seq
.....................................................aacuguacaaccuucuagcuuuc........   2    0   seq
.......................................................cuguacaaccuucuagcuuu.........   3    0   seq
.......................................................cuguacaaccuucuagcuuuc........   10   0   seq
.......................................................cuguacaaccuucuagcuuucc.......   30   0   seq
.......................................................cuguacaaccuucuagcuuuccu......   3    0   seq
........................................................uguacaaccuucuagcuuucc.......   2    0   seq
........................................................uguacaaccuucuagcuuuccu......   6    0   seq
```
